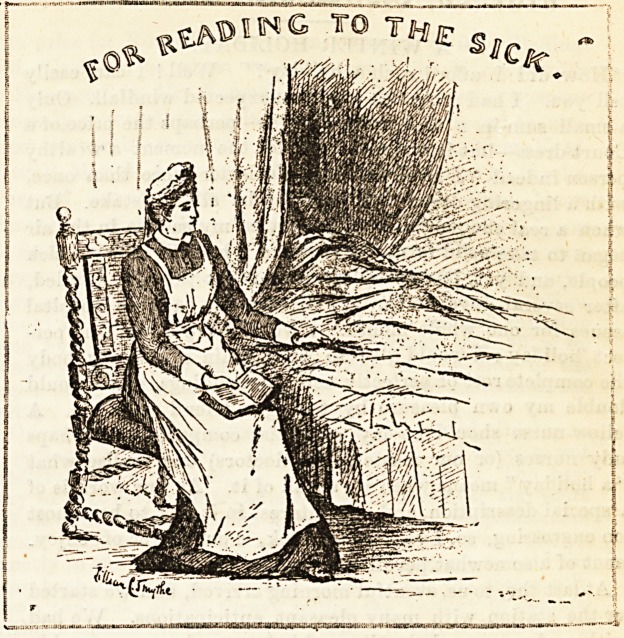# Extra Supplement—The Nursing Mirror

**Published:** 1890-12-20

**Authors:** 


Being the Extra Nursing Supplement of "The Hospital" Newspaper.

All Contributions for this Supplement should be addressed to the Editor, The Hospital, 140, Strand, London, W.C., and should have the word "Nursing" plainly written in left-hand top corner of the envelope.

### En Passant.

Rural Nursing Association.—A meeting in support of this association was lately held at Exeter, the Lord-Lieutenant of Devon presiding. The audience was large and the proceedings enthusiastic, the result being the establishment of a branch of the association for the county of Devon. Lady Poltimore has been appointed president, Miss Sanders hon. secretary, and Lady Clinton is on the Committee. The sick of Devon will soon have sturdy nurses tramping over their moors, and teaching in poor homes how best to tend the sick to ensure comfort and recovery.

Ward Maids.—The Middlesex Hospital has just commenced ward maids: the London Hospital proposes to augment its ward maids. At King's College Hospital the ward maids live in the building, have a uniform, and are paid 7s. 6d. a week. This is the ideal arrangement of the sort, but is not always possible to hospitals in crowded towns. Guy's has 24 resident ward maids, and about 20 non-resident charwomen. The wards at St. Bartholomew's are scrubbed by contract, and there are 28 "ward assistants." The new ward maids at the Middlesex are to have a uniform, but they are obliged to be lodged outside the building; this is also the case with the ward maids at the London, who are paid 10s. a week and get their dinner. These facts should be interesting to matrons who are trying to reduce the menial duties which nurses have to perform in some of the provincial hospitals.

Strong and True.—"The mischievous zeal which inspired a few meddlesome busy bodies to attack the management of the London Hopital has by this time pretty well exhausted itself; and the remarks made by the Chairman at the meeting of Governors will be endorsed by all sensible persons. The London Hospital is as well managed as most other institutions of the kind; and the charges of maladministration, especially in regard to the treatment of nurses, are groundless. The system may not be absolutely perfect; but even within the last decade there has been a marked improvement, and there is not the slightest reason why the public should not continue to feel the fullest confidence in tce energy and discretion of the governors. It is monstrous that the work of a great charity should be hindered and harrassed by irresponsible criticism in which ignorance is mixed with malevolence."—*St. James's Gazette.*

A Nurse for Buxton.—It has been decided to start a district nurse at Buxton, and the expenses of the first year have already been subscribed. At the inaugural meeting Mr. F. Turner said that, having been the parish doctor for some twenty-five years, it occurred to him that he might be of some assistance in helping them in their deliberations. He had seen the poor in their distress, and could speak from experience. They did require such assistance as it was proposed to render. They were many poor people who were perfectly incapable of rendering that nursing assistance to their neighbours that the Doctor and the ladies before him would wish they should receive. This movement would also do good, because it would bring the ladies more into contact with the poor. A great deal, however, depended upon the manner in which this undertaking was carried out. It should be undertaken very carefully at first, not allowing too great expectations. He had great pleasure in moving "that it is desirable that a district nurse be appointed for Buxton and surrounding district, the district to be comprised within a radius of two miles from the Buxton Post Office." The resolution was carried, and a committee of ladies appointed.

Short Items.—The Rev. E. P. Gregg and Mr. W. M. Baynes, lately gave a limelight exhibition to the patients and nurses of the Torbay Hospital. Miss Schofield undertook the arranging, and a very happy evening was spent by all.—A testimonial is being subscribed to for Nurse Hart, who has served the Luton Cottage Hospital well for 18 years.—The Brassey Holiday Home has received two Christmas gifts already—a cheque for £20 and some grouse. Miss St. Quintin writes to the *St. Leonard's Observer* in praise of the home.—Twelve attendants at Broadmoor Asylum have taken the certificate of the St. John Ambulance Association.—Nurse Deas, of Barton-on-Humber, has visited 132 patients during the last year; there were 23 deaths and 79 recoveries.—We learn from the *Queen* that Miss J. Burrows has been appointed head nurse of the Wakefield Riding Asylum. Why do not asylum matrons send us notice of their appointments as hospital matrons do?

The Fate of Reformers.—Before the Braintree Board of Guardians, on December 1st, was a long report from the chaplain, including a tabulated statement, showing that one paid nurse had charge of between 60 and 70 beds, which were located in a dozen different wards, beyond earshot of each other. Under the nurse were some ten or a dozen pauper assistants, either deficient in mental, physical, or moral strength. The chaplain began to point out at length the evil of this system. Mr. Southcott: I rise to move the closure in reference to this matter, and that we proceed to the next business. Mr. J. Polley: I will second that. The Chairman said the chaplain had certainly outstepped his province in the matter. Mr. Southcott's motion was carried *nem. con.* Now we are not sure whether the chaplain was foolish in being long-winded, or whether he spoke without giving previous warning, but it is notable that at present his facts have been undisputed. Louise Dovey, of the Thanet Union, has just been appointed nurse at Baintree; it would have been a good chance for the Guardians to see to a few improvements in the nursing.

Fever Hospitals.—The following resolution has been passed by the Committee of the Stoke Newington Ratepayers' Association: "That this meeting, having considered the statements of Nurse D. Halkin and Mr. Simpkin respecting the diet and treatment of patients at the Homerton Fever Hospital, is of opinion that a public inquiry is imperatively required." A writer, who does not wish her name published, sends us a long letter, showing the opposite side of view to that shown by Nurse Halkin; we have no doubt this writer has much on her side, for there are always two sides to a question, but still the weight tells with the woman who signs her name. "You are always hard on fever hospitals," writes another correspondent, and so we feel bound this week to say a good word for the North-Western Hospital at Haverstock Hill. There is a medical superintendent and two assistant doctors; the patients speak highly of the care they receive; they also have a good word for the food, especially the beef tea. The nurses have quarters quite away from the wards, there is a sitting-room containing books and a piano. Quite a large number of patients at Haverstock Hill are little children, and they thoroughly repay, by their pretty affection, the careful tending of the nurses. It is far from our desire to speak ill of any hospital, but the day comes sometimes when it is impossible to keep silence, and we heartily back the ratepayers of Stoke Newington in their call for a public inquiry, being sure that such a step will end in the Homerton Hospital taking a worthy place amongst its compeers.

### Lectures on Surgical Ward Work and Nursing.

By Alexander Miles, M.B. (Edin.), C.M., F.R.C.S.E.

### Lecture VII.—PREPARING FOR AN ORDINARY DRESSING.

Having last week indicated the various materials and utensils in every-day use, and some of the more important practical points in connection with each, it may be convenient that I should recapitulate in a tabular form the furnishings of a ward table and dressing tray.

*Ward Table and Dressing Tray.*—The arrangement of these is so much a matter of individual taste, that one cannot lay down rules with regard to it. On the ward table should be found a Winchester jar of carbolic lotion, 1 in 20; a Winchester jar of corrosive lotion, 1 in 1,000; a Winchester jar of corrosive lotion, 1 in 2,000; a 20-ounce jar of corrosive lotion, 1 in 500; a Winchester jar of boracic lotion, saturated. All these jars should have glass stoppers, and be carefully labelled. They should all be marked "Poison," and must not be within the reach of children. Eucalyptus oil; unguent for urethral instruments; vaseline. These should be in wide-mouthed glass bottles, containing about four ounces of each. Iodoform; boracic powder. These should be in wide-mouthed glass bottles, covered with fine gauze firmly fixed on with an indiarubber band, for dusting. Drainage tubes, pads of plain gauze; antisepticised safety pins. The tubes and gauze are best kept in wide glass jars, about six inches high and four inches in diameter; the pins (to be used only for transfixing drainage tubes) in a small glass bottle with a stopper. The carbolic lotion in which all these are kept should be frequently changed, as it loses in strength by volatilising. A small box containing sulphate of copper (blue stone). In addition, there should always be at hand four to six lotion basins of different sizes, plain and enamelled, two kidney-shaped basins, two bleeding cups, two solution tins, one leg tray, one dirty-dressing tray, one pail with close-fitting lid for hot water, one pail to hold dirty lotions, &c., one Higginson's syringe, one brass or glass syringe.

*Dressing Tray.*—This may be of wicker-work, or, better still, of tin, about one and three-quarters to two feet long, by one foot broad, and about two inches deep. It should contain a supply of plain surgeon's lint, boracic lint, protective, gutta-percha tissue, gauze bandages (carbolised and double cyanide), domette bandages (plain and sal alunbroth), linen bandages (plain), adhesive plaster, corrosive wool and wood wool (in tin boxes with close-fitting lids), safety pins, a pair of scissors, a pair of dressing forceps, a pair of sinus forceps, a pair of dissecting forceps, a probe, and a measuring tape.

*A Simple Antiseptic Dressing.*—Having now considered the general principles of antiseptic surgery, and the means at our disposal of applying these principles, we shall go on to study the practice itself. It will often be your duty to prepare a patient that the surgeon may dress him at his ward visit, and this includes not only the preparation of the patient himself, but of all the dressings, lotions, etc., that may be used in the process. There is no way in which a nurse can show her capabilities better than in the performance of this apparently simple duty. She must take every precaution that the patient is not unduly exposed, or wearied more than is absolutely necessary. She must also make certain that she has everything at hand that can possibly be wanted, so that there will be no delay during the dressing by things having to be sent or searched for. She must carefully watch the operation as it goes on, and *anticipate* the surgeon's wants. This "faculty of anticipation" is one which is much appreciated by surgeons in their nurses. It should be the aim and ambition of a surgical nurse never to require to be asked for anything during a dressing, but always to have the required article ready to the surgeon's hand just at the moment it is needed. This is of importance to the patient as well as to the surgeon, because many people, and especially women and children, are much more excited and frightened than pained by being dressed, and this excitement and fear is increased if there is a constant conversation going on between the surgeon and the nurse as to what is being done. The less talking, then, that goes on at the dressing the better. If by any chance the nurse should have forgotten something, and should notice her mistake just when the dressing is beginning, she should take some opportunity of going to rectify it when her services are not required. She should not rush off immediately she notices the omission, just, perhaps, when she is wanted to hold a limb, or remove a bandage, but should wait till some part of the dressing which will engage the surgeon's attention for some little time, and then she can quietly and quickly go, her absence, perhaps, never being observed. I well remember a probationer who had this faculty of anticipation in a marked degree. I have dressed with her nearly a dozen cases daily for several months, and can scarcely remember having had to wait for anything all that time.

What, then, are the main points to be attended to in the performance of an ordinary simple antiseptic dressing?

(1) *The comfort of the patient.*—This must always be your first care, as it rests entirely with you.

(2) "*Antiseptics.*"—This includes your own personal "surgical cleanliness," that of the surgeon, and of the dressings.

*To prepare the patient.*—Let us suppose that the wound to be dressed is that resulting from a severe crush of the leg below the knee, and that the spray is not being used. Protect the patient from draughts, as well as from the gaze of other patients in the ward, by bringing the ward screens round his bed. You should be careful in doing so to leave plenty of room all round the bed, so that doctors and nurses may move about without knocking against the screen. Have the screen firmly standing. I have seen a patient start so violently on a nurse knocking against a screen as to do himself serious harm. Try to arrange the doorway between the screens so that those inside may reach it from either side of the bed without passing one another. The most convenient place is usually opposite the foot of the bed. You must also see that no more of his body is exposed to the air than is absolutely necessary, especially in cold weather. Arrange the bed clothes so that only the injured limb is uncovered, and only as much of it as is sufficient to render access easy.

*Protect the Bed.*—This you will do by covering as much of the bed as comes within the area of your operations with macintosh waterproof. You will find it an advantage to fold up the edges of this all round so as to form a kind of gutter, so that any lotion spilt on to the macintosh will not find its way on to the sheets. The same end may be attained by sewing a roll of wool into the edge of the macintosh all round, thus forming a thick border.

*Carbolised Towel.*—Over the macintosh you spread a "carbolised towel," that is, a towel which has been soaked in 1 in 20 carbolic and wrung out. The object of this is to have an antiseptic surface next your wound, so that if by any chance the wound comes in contact with the bed, septic contamination is rendered impossible. Instruments, sponges, etc., can with safety be laid on this dipped towel. Never use a towel with holes in it for this purpose. The precaution of the dipped towel is one which is not always taken, even in hospitals where the antiseptic system is supposed to be carried out in all detail. I have even seen a surgeon open an abscess with nothing but a macintosh on the bed, and from this he lifted his instruments, sponges, and drainage tubing. Now no one supposes that a macintosh is antiseptic, or even aseptic, and it seems unreasonable that a wound should be douched with carbolic lotion and dressed with antiseptic wool while such a simple and obvious precaution should be so often neglected. The towel has another advantage, namely, that it absorbs a considerable quantity of the lotion which runs off the wound, and so prevents soiling of the sheets. From a nurse's point of view this is a sufficient reason for her using it even should she feel that it is not her place to suggest the antiseptic precautions to her chief. When the leg-tray is used, the macintosh should be laid under it, and the carbolised towel over it, so that by the former the sheets will be protected, while the latter will secure an antiseptic surface next the wound, without interfering with the escape of lotion into the tray.

(*To be continued.*)

### Nursing Medals and Certificates.

No. II.—THE CHARING CROSS.

Until last year the nursing of Charing Cross Hospital was in the hands of the Sisters of St. John's House, who had a badge of their own we hope to reproduce later. But in 1889 it was resolved to introduce lay nursing at Charing Cross, and the formation of a new body of nurses was entrusted to Miss Hughina Gordon. The present system is similar to that at St. Thomas's, for Miss H. Gordon received her training from the Nightingale School; the nurses serve for three years, and then receive a certificate as follows: "(Name) —— has during the period above stated given satisfaction in her service as nurse, the particulars of which are recorded in the registers of this institution in the possession of the Lady Superintendent, to whom all reference may be made." This cautiously worded document is signed by the treasurer only; there is nothing superfluous about the Charing Cross Hospital certificate, and its sole ornamentation is a small sketch of the building at the top.

But the nurses have a medal which they receive when they sign after their month of probation; it is a bronze representation of the cross known as the "Charing Cross"; it is Maltese in shape with *fleur de lis* between the arms; the name of the hospital is inserted in blue enamel. The nurse wears this cross slung round her neck whenever she is in uniform; if she leaves before her agreement is up she forfeits her cross, but if she completes her training the medal is hers for ever.

The sisters of the institution wear a similar cross, only made of silver; the Lady Superintendent also wears the silver cross, but she pins it on the left breast instead of wearing it round her neck. Of course, the new training school at Charing Cross has been in existence such a short time that its badge has not yet come to be generally known; and be it remarked that the cross is solely a badge and in no way a reward of merit. In the days to come we expect the pretty cross will be worn by those who will be scattered all the world over, and it will be regarded as a most precious possession and a mark of honour.

---

A Good-natured Tempest.—It was stated in the *Echo* that, during the late storm, a brig "brought into Dover harbour two men with their ribs and arms broken by a squall off Beachy Head. The deck house and steering-gear were carried away, and the men taken to Dover Hospital." Who shall say after this that storms do not temper severity with kindness? This particular one, it is true, broke some ribs and arms, and carried away portions of a brig, but, in the very act of doing this it took the sufferers and laid them, apparently, on the steps of Dover Hospital. If we must have storms, may they all imitate this motherly example.

PREPARING FOR CHRISTMAS.

Christmas is once more close upon us, but a few days and we shall have entered into that season of joy and festivity, for which most of us are making some sort of preparation. Each person is doing so according to his taste. The young are getting ready their skates, for there has been already ice and snow, and it promises to be an old-fashioned winter, while for the same reason the old are supplying themselves with stores of warm garments. The shops are full of food and clothing, toys, books, fruit, flowers, and pictures; in short, everything bright and attractive to the eyes of the purchaser.

Families who have been separated by circumstances will soon meet together, presents be exchanged among friends, and, let us hope, all jealousies and heart-burnings laid aside, for is it not the season of peace and good-will? And all these things going on are preparations for keeping Christmas. May we all have a very happy time! But how, and in what way, that is a question best answered by our joining together with one accord, old and young, rich and poor, one with another in praising God and helping our neighbours in love.

I am afraid human nature now is very much like what it was eighteen hundred and ninety years ago. Men were going on then eating and drinking, and marrying and giving in marriage, while the most wonderful thing was happening in a poor little city of Syria. Nobody was expecting the birth of Christ the Lord. He was born at Bethlehem, in a stable, and laid in a manger. Only a few wisehearted men knew of it, because they had watched the signs of heaven. And we are going on just the same at this time. The air is full of the glad sound at Christmas-tide, which tells us of the birth of a Saviour, and we turn away to listen to the sounds of worldly mirth and revelry. Do not let the sounds of earth drown the notes of heaven. But harken to what they sing above. They tell us glad tidings, glad tidings of great joy—that the Saviour is coming who will make His home in all meek and contrite hearts, that He will free the prisoners of sin, that He will bind up the broken-hearted, that He will staunch the bleeding soul,

"And with the treasures of His grace
Will bless the humble poor."

We will prepare this year for His coming by turning our voices to join in glad Hosannas to the Prince of Peace, and make a throne in our hearts where He may sit and reign over our thoughts and words and actions evermore. We will turn out all impure thoughts and unkind feelings against our neighbours and friends, and keep our minds full of love and gratitude for the mercies, bodily and spiritual, which God sends us at this time.

### Nurses on Their Travels.

### "A WINTER HOLIDAY."

"How did I afford such a holiday?" Well! I can easily tell you. I had a legacy, a most unexpected windfall. Only a small sum in a rich woman's eyes—perhaps the price of a Court dress—but it made me feel for the moment a wealthy person indeed. I read the lawyer's letter more than once, with a lingering dread that it might be all a mistake. But when a real cheque followed the letter my castles in the air began to take solid form. For years I had lived with sick people, and yet, loving my work amongst them, I decided, after several days' hesitation, to turn my back on hospital scenes for one whole month. I would have a real and perfect holiday; I would give to tired brain and weary body the complete rest of perfectly new surroundings, and I would double my own pleasure by taking a friend with me. A fellow-nurse should be my congenial companion. Perhaps only nurses (or our masters, the doctors) can realise what "a holiday" means when we speak of it. As our work is of a special description, and our interest in it apt to be almost too engrossing, so, I venture to think, is our power of enjoyment of a somewhat peculiar intensity.

At last the, to us, eventful morning arrived, and we started for the station with many pleasant anticipations. We had neither of us crossed the Channel before, and were agreeably surprised to find ourselves good sailors.

A night in Paris, and a tiring day spent in seeing some of the sights of that gay city, then a hasty meal before beginning, in an evening train, the long journey to Bayonne. The season being winter, we naturally turned towards the "Sunny South."

Already we were enjoying the brightness, the novel costumes, and the vivacious chatter of the French, which had attracted us directly we landed—but even these things at last lost power to make us forget our weary bodies. Towards midnight, as we travelled on through the now "silent land," a grave doubt of the delights of travelling began to assail me; but these doubts vanished with morning light, and never returned.

The abominable economy in space practised on French railways, where the filling up of each seat is rigidly adhered to, did not commend itself to us. After a night spent in sitting upright in a more or less cramped position, how gladly we hailed daylight and our half-hour's halt at Bordeaux!

A rapid breakfast and an equally rapid toilette sent us cheerful and refreshed to secure our seats for Bayonne.

The railway now traversed the far-famed "Landes," and a very curious and mysterious country it looked in the early morning. "So lonely!" was my first thought, "so silent!" was my second.

A shepherd on his stilts was a sight we grew watchful for, but the solitary figures hardly lessened the feeling of almost oppressive loneliness.

By-and-bye we had our first sight of the glorious Pyrenees, and then, indeed, all recollection of aching backs and weary heads vanished away.

We found the quaint town of Bayonne full of interest—first the cathedral, then the camp, the river and harbour, the picturesque rows of shops and stalls, all so completely un-English to our observant eyes. We were glad to retire early to our inn, and secured a long, as well as a very sound, night's rest, for truly we "slept the sleep of the weary."

(*To be continued.*)

---

"Now, be careful how you drive, cabby, and go slowly over the stones, for I hate to be shaken. And mind you pull up at the right house, and look out for those dreadful railway vans." Cabby: "Never fear, sir, I'll do my best. And which 'orsepital would you wish to be taken to, sir, *in case* of an accident?"

### Everybody's Opinion.

[*Correspondence on all subjects is invited, but we cannot in any way be responsible for the opinions expressed by our correspondents. No communications can be entertained if the name and address of the correspondent is not given, or unless one side of the paper only be written on.*]

### ASYLUM ATTENDANTS.

"C. E." writes: I should be glad of the opinion of your readers on the following notice. Such a notice would not be tolerated by a hospital nurse for a moment I am sure. "Durham County Asylum.—To the attendants and nurses:—I am always glad to assist attendants and nurses in procuring better situations, but I cannot approve of their coming here, learning their duties at the expense of the asylum, and then going to other asylums to fill similar situations. I make it a rule never to take attendants or nurses from other asylums, and I shall do all I can to discourage nurses and attendants being received from this into other asylums. I do not think it is for the good of any of the institutions that such a practice should be made. I therefore beg all attendants and nurses to understand that if application, even with my sanction, is made for similar appointments in other asylums, it will be tantamount to a month's notice to leave.—Robert Smith, M.D."

### THE DIET OF NURSES.

Miss Twining writes: As you have noticed my opinion expressed in the paper read by me in 1885, with regard to the diet of nurses, will you allow me to say that it may be misunderstood unless I state the quantity of meat to which I objected (in the Kensington Infirmary)? I found that the allowance of one pound daily was more than could be consumed, and was therefore wasted. The unanimous wish of the nurses was to have less meat and more puddings (which were not supplied daily). The allowance was therefore reduced to three-quarters of a pound, and the saving effected enabled us to add the desired puddings and tarts. I still consider that when appetites are capricious or failing (as who can wonder they are?) soup is a most desirable and nourishing food, and it is surprising how seldom it is supplied. May I add one word to your correspondent "A. E. S."? It is strange to find that it seems to be always supposed that women will do harm by interference, as Mrs. Jameson remarked thirty-five years ago; but if matrons are perfectly able and willing to provide everything necessary for the nurses' comfort, it would seem that committees of management (all of whom are, or ought to be, "overlookers") are quite unnecessary, whether composed of men or women. Perhaps the whole question is begged in the words "A matron who cares for her nurses." I willingly admit that matters have improved considerably since I gave my facts five years ago, but that is not saying we have reached perfection.

### ATTENDANTS AT BERRY WOOD.

Miss Pauline Buckland writes: It is now some time since I contributed to your paper, and knowing the interest you take in nurses of all kinds, it is with pleasure I write again. In my last letter to you I stated there were classes held here in which the nurses received lessons on various subjects, such as circulation, respiration, &c. The lessons were continued, and we kept on advancing, and found the more we learned the greater our interest became. For the last two months, however, our attention has been given entirely to ambulance work, and I am very pleased to be able to say eleven attendants and sixteen nurses (all who entered) passed the "first aid" examination, which was held in Northampton on the 17th of last month. The attendants were carefully taught by Dr. Greene, and the nurses by Dr. Harding. Perhaps some may think, and say, there is no great honour in obtaining First Aid Certificates. Well! I admit that myself, still there must be a commencement to all things. We intend, if possible, to persevere, and I trust, when we finish, some of us, if not all, will have earned the highest honour, viz., St. John's Ambulance Medal. We are resting now from nursing classes, and are taking part in a comic opera, called "Bluebeard," for the amusement of the patients. We find it a most agreeable change, and enjoy the rehearsals equally as much as we did the classes. The doctors instruct us just as kindly and untiringly in theatricals as they did in nursing.

### A WEDDING GIFT.

MISS EMMA DURHAM writes from Farringford, Freshwater, Isle of Wight: I see in the *Times* newspaper that Prince Aribert of Ainhalt has become engaged to Princess Louise Augusta, daughter of the Prince and Princess Christian of Schleswig-Holstein, and the thought has struck me, and doubtless it will many others, that we English nurses might subscribe for a wedding gift, to show our gratitude and admiration for the princess's mother, her Royal Highness Princess Christian, who has always taken such a kindly interest in nurses and nursing.

### NURSES' HOURS.

"A. E. S." writes: May I make a few remarks on Mr. Burdett's speech, "Nurses' Food, Work, and Hours of Recreation"? All he suggests can be, I have no doubt, well carried out in a London or large provincial hospital; but it would be impossible to work a small provincial hospital, say 50 beds, in the same way. Where there are no sisters, assistant nurses, or ward maids, and where all the work—washing up, cleaning up fire-sides, &c.—is done by the probationer, and where, when the charge nurse is off duty, the probationer must of necessity take her place. There is more work in two or three small wards than in one holding the same number of beds, and where patients can all at one time be under the eye of the nurse. It has been tried to make a rule for two hours off daily, but it has failed, and now three hours three times a week is found to answer better, and half a-day once a fortnight. The night nurses change every six weeks. Provincial nurses get a very good individual training as there are no students, and they are well looked after, and each nurse must be more thoroughly known than in a larger place. Gladly would all holidays be lengthened to a month (two weeks at separate times), but the expense of an extra helper to the hospital and two journeys (often long ones) are great considerations. People will, we hope, take the hint, and give more little treats to nurses, who have no moneys for boats, drives, concerts, &c. It is a fact that the nurses in our hospitals receive more consideration in words than in deeds, and an afternoon tea, a supper, or even a cake, are things much appreciated and long remembered. When will people cease the "romantic rubbish" that goes on about nurses, their dress, their love for their patients and work, and remember that hundreds of us took up the work because of our "daily bread," and though, as I have before said, there is no constant talk of "loving" our work, we do it kindly and well, look after food and comfort for all sorts and conditions of men and women—nurses, patients, and staff.

"MATRONA" writes: Why all this hubbub and nonsense about nurses feeding and resting before entering for their full training? There is a trial time. Why don't those who think so much about eating and playing give it up, and make room for others, who would be ashamed to have so much to say about institutions where they find employment, and against the hard worked matrons who, I believe, never fail to try and make things comfortable as circumstances will permit? Here is a tale told me by a matron a few days ago: A new night-nurse arrived, and on the matron visiting the wards last thing, the nurse informed her that she should like a chop cooked during the night. You see, sir, what things are coming to. Night cooks will be required soon, as well as night nurses. Do you know is cooking done at night in any hospital; and, if so, by whom? And will you also tell me, please, what you think of the appended dietary for nurses: Breakfast at 6.45, tea, coffee, or cocoa, bread and butter, eggs fish, or bacon; lunch at 10, coffee or soup, bread and butter; dinner at 12.30, meat (varied), chiefly beef and mutton, twice a-week two vegetables, twice a-week soup, twice a-week pudding; tea 4.15, tea, bread and butter; supper 9, cold meat, or fresh-cooked fish?

### Notes and Queries.

### Answers.

*Lady Roberts' Fund for Nurses.*—All applications of nurses in respect of employment under this fund should be addressed to the Under Secretary of State, India Office, Whitehall, S.W.

*The Countess of Dufferin's Fund.*—All communications should be addressed to Colonel J. Robertson, C.I.E., 27, Inverness Terrace, Hyde Park, W.

*Subscriber.*—We never prescribe; consult a medical man. A nurse's remedy for sore feet is generally rubbing on methylated spirit.

*A Trained Nurse.*—Your letter is libellous; we cannot print it. This is a pity, as we should like a calm, authentic account of your view of the Homerton Hospital.

*A Fellow-Worker.*—*F. T. Paris, and others.*—Your letters have been unavoidably held over till next week.

*Sister.*—A correspondent would send you six copies of a little book called "Daily Light on the Daily Path" for your patients who have no Prayer-Books. Please let the Editor know if you wish for them.

*F. R. Jarvis.*—Write to the local secretary of the St. John Ambulance Association (Mr. Charles H. Green); or doubtless Dr. Damer Harrison or Dr. Whitford could tell you if there are any classes now being held.

### Examination Questions.

THE prize for November has been awarded to Mrs. Bedingfeld, Glasbury, to whom we have sent "The Nurse's Guide to Massage," by Mr. Samuel Hyde. The following answers so nearly approach the prize answer, we feel bound to give them honourable mention: Nurse Ethel Wilson, Nurse E. M. Bontell, Miss A. Anderson, Miss M. Gough, Nurse M. Stocks, Nurse M. Sewell, Nurse Whiteman, Nurse A. Price, Nurse Henderson, Sister Mary Gardner, Mrs. F. E. M. Hart, Nurse E. Andrew, and Miss Lily James. All these answers were correct and full, and differed but slightly from the prize answer given below. The question for December is:—"How should a cold bath be given if ordered for a patient suffering from hyper-pyrexia?" Answers must reach this office by January 10th; they must be written on one side of the paper only, and must bear the full name and address of the writer.

QUESTION FOR NOVEMBER.—How would you administer to a patient (1) castor oil, (2) cod liver oil, (3) effervescent medicine, (4) a powder, (5) a pill? *Answer.*—1. The best way to give castor-oil to adults, and children over six years old, is to put in a medicine glass one or two teaspoonfuls of lemon juice to the same quantity of water. Pour the oil exactly in the middle of this. Direct the patient to hold the head back and the mouth well open. Pour the dose gently down the back of the throat, telling the patient to swallow without closing the lips on the glass. If this is done the oil will slip down followed by the lemon juice, and no taste of the oil or greasiness of the mouth will be perceived. Moreover, when given on the acid, the oil is less liable to "repeat" than when given in other ways. Brandy may be used instead of lemon, and in cases in which neither is permissible, milk, warm or cold, may be substituted. For young children castor-oil is best mixed with hot milk, by putting the oil and milk into a small bottle, and shaking them till they are thoroughly incorporated. A little sugar and a few drops of cinnamon water may be added. In the case of infants I find it best to dip a teaspoon into warm water for a few seconds. Pour in the oil, and holding the mouth open with a finger of the left hand, place the spoon on the tongue, rather backward, and let the oil slip gently down. The spoon should not be removed till the oil is all swallowed. Oil can also be given to children in a spoon well powdered with sugar. In all cases avoid making the dose too bulky. 2. Cod liver oil should be administered in the same way as castor oil, the medium being either orange or lemon juice, ginger or orange wine, milk, or merely water. Many patients get so fond of cod liver oil that they prefer taking it in its natural state, or flavoured with a pinch of salt. 3. Effervescing medicines are tiresome to give to a patient who is very ill, especially if the dose is large. In these cases the effervescence should be allowed to subside slightly before the medicine is given, and care should be taken to better support the patient with pillows, or to raise the head on one arm passed under the pillow. A cloth should always be placed under the chin in case the draught should bubble over. The tumbler used should be large, a soda-water tumbler is best, or a feeding cup or "moustache" cup may be substituted. Have the alkali in one cup, the acid in another, and pour the two together when the patient is quite ready. In all cases, whether the draught be large or small, care should be taken that it does not spurt over the patient's face. Seidlitz powders are often more efficacious if mixed with warm water, and ten to twenty drops of essence of ginger added makes them more palable. 4. A powder if small should be placed on the back of the tongue, and a little water or other liquid immediately swallowed. Or it may be placed between two thin pieces of bread and butter, or bread and jam. This is a good way of giving it to children. Or it may be mixed in a little jam, honey, or a spoonful of gruel. If mixed in a thin liquid the powder is apt to sink to the bottom and be partly lost. 5. A pill should be thrown well to the back of the throat, and some water swallowed at once. Or it may be taken in a little jam. Some people fail entirely to swallow pills. When this is the case, the pill should be crushed and treated as a powder. It should be remembered in giving all nauseous drugs that the further the medicine is placed to the back of the tongue the less will the taste be perceived. The mouth should be rinsed out both before and after the medicine."

### On £30 a-Year.

There used to be amusing articles in women's papers on such subjects as "How to Dress as a Lady on £5 a-year," and the public greeted these articles with jeers. Some nurses seem to consider the problem of living on £30 a-year equally impossible, but it can be done. I grant it is a poor reward for a life of labour, and the young nurse ought to secure a higher pension than this, but some of us older nurses are glad enough to be able to get £30. There was no Pension Fund in our young days.

I live with my niece just outside a county town, in a small cottage containing two sitting rooms and a kitchen down stairs, and three bedrooms upstairs; there is a nice little flower garden at the back. The rent is 5s. 6d. a-week and no taxes. I pay the rent, which amounts in all to £14 6s. a-year, and I also make a present to my niece either in goods or money, of about £1 every quarter. The houses are chiefly inhabited by mechanics, but that does not affect us. My niece makes shirts; she suffers from deafness, which precludes her from earning her living otherwise, though she is well educated. She makes from £60 to £70 a-year, and is a very clever and shrewd housekeeper and a good cook; we therefore live in comfort. Though I am well over sixty, and somewhat crippled with rheumatism, I make all the button-holes for my niece.

I furnish my bed-room and make some additions to the sitting-room. My laundress and newspapers amount to £3 a-year; my clothing costs me £6 a-year, and I wear wool summer and winter, and it lasts a long time. I confess to using two bottles of brandy a year, and I take half a tumbler of beer with my dinner. For my supper I usually have bread and milk. The brandy I pay for myself, but my niece boards me otherwise in consideration of the fact that I pay all the rent. She calculates that she does not lose by the transaction. If my friends want to see me they have to pay my railway fare or else come and call on me, for travelling is an expense I cannot meet. I keep an exact account of all my expenditure, and last year I had 7s. 8d. to carry over. When I first had my annuity I boarded and lodged myself on the £30, and managed very well, never being without the orthodox threepenny-bit for church collection, or for anyone worse off than myself.

My expenses now then may be summed up thus:—

| | £ | s. | d. |
|---|---|---|---|
| Rent ... ... ... ... ... ... ... | £14 | 6 | 0 |
| Goods given ... ... ... ... ... ... | 4 | 4 | 0 |
| Furniture ... ... ... ... ... ... | 0 | 15 | 0 |
| Dress ... ... ... ... ... ... ... | 6 | 0 | 0 |
| Laundress, stamps, and newspapers ... | 3 | 0 | 0 |
| Brandy ... ... ... ... ... ... | 0 | 12 | 0 |
| Total ... ... | £28 | 17 | 0 |

Which leaves me 6d. a week in my pocket for sweets, fruit, or any other little fancy, or for my favourite charity. I only reckon my laundress at £2, as I can do small things at home very often; I take two weekly newspapers at 1d. each, and rarely I buy a daily paper if there is anything exciting going on. Stamps and writing paper certainly do not cost me more than 10s. a year.

There is no hardship in this life at all; it is pleasant and peaceful; of course, a confirmed grumbler or one without the sustaining power of religion might find cause for grief in the fewness of the spare coppers, but having good food and raiment I am therewith content. But one thing I would point out to young nurses, that it is not for them that I have written this short account of my expenditure, but for my compeers; because life is pleasant on £30 a-year, that is no reason why those who can afford it should not secure a pension of £60 a-year.

### Appointment.

[It is requested that successful candidates will send a copy of their applications and testimonials, with date of election, to The Editor, The Lodge, Porchester Square, W.]

Derby General Infirmary.—Miss Frances Webster-Wedderburn has been appointed night superintendent at this infirmary. Miss Webster-Wedderburn has just finished her three years' service at St. Thomas's; for a month she worked at Derby on trial, and now, in spite of the present discomforts at that infirmary, she has decided to accept the above permanent appointment. Her testimonials are excellent.

### To "The Hospital" Supplement.

"Nursing Mirror," clearly showing,
Pleasant reading, pages bright,
Prudent counsel, gentle warning,
Nothing that need fear the light.
Peace, content, forbearance, union,
Nursing news from all the land;
Helpful thought sent forth, and learning
Reflected to our nursing band.

"Nursing Mirror," clearly showing
Our need of guidance; here we see,
Heedless words will often injure,
Slanders never ought to be.
Queries asked, and queries answered,
Letters penned by many a hand;
Opinion, grievance, fact, and fiction,
Reflections from our nursing band.

"Nursing Mirror," clearly showing,
England's nurses heed each call—
Burning fever, deadly pesthouse,
Many even martyrs fall.
Children nursed with love unfailing,
Thus helping waifs to understand
The glorious precept, "God is love,"
Reflected by our nursing band.

"Nursing Mirror," like a beacon,
Clearly pointing out the way,
Gave to us a valued guidance,
Showed us where the dangers lay.
"God grant" (thus shown) we go aright,
Faithful work with heart and hand;
Adding to the lustrous light,
Reflected by our nursing band.

W. B. H.

### Amusements and Relaxation.

**N.B.—THIRD QUARTERLY WORD COMPETITION**

**Commenced Oct. 4, 1890; ends Dec. 27, 1890.**

Three prizes of 15s., 10s., 5s., will be given for the largest number of words derived from the words set for dissection.

N.B.—Word dissections must be sent in WEEKLY not later than the first post on Thursday to the *Prize Editor*, 140, Strand, W.C., arranged alphabetically, with correct total affixed.

The word for dissection for this, the TWELFTH week of the quarter, being "GOOD CHEER."

| Names. | Dec. 11th. | Totals. | Names. | Dec. 11th. | Totals. |
|---|---|---|---|---|---|
| Jenny Wren | 30 | 622 | Checkmate | — | 76 |
| Tinie | — | 55 | Silver King | — | 163 |
| Agamemnon | 34 | 640 | S. Anthony | — | 76 |
| Patience | 36 | 639 | Quackah | — | 75 |
| Ecila | 34 | 640 | Reynard | 35 | 582 |
| Lightowlers | 34 | 612 | Sally | — | 27 |
| Rouge | — | 89 | Success | — | 61 |
| Wyamaris | 37 | 630 | Caledonia | — | 52 |
| Qu'appelle | 35 | 539 | Nurse Emma | — | 305 |
| Nosam | 35 | 568 | Hazel | — | 20 |
| Nurse Hilda | — | 44 | Pallas | — | 48 |
| Lady Betty | 34 | 586 | Puss | — | 15 |
| Grenelle | — | 43 | Shakespeare | 35 | 525 |
| Daisy | — | 324 | Melita | 30 | 566 |
| H. A. S. | — | 157 | Nora | — | 10 |
| A. B. C. | — | 66 | Elsie | — | 35 |
| Liz | 27 | 444 | Espèrance | — | 27 |

### SPECIAL NOTICE TO CORRESPONDENTS.

N.B.—The word dissections of "Good Cheer" must reach the office not later than the first post on Wednesday morning next, December 24th.

## Figures and Tables

**Figure f1:**
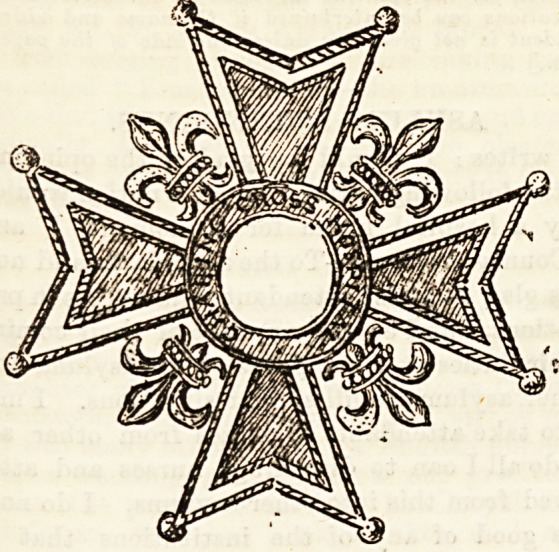


**Figure f2:**